# Left Lateral Segment Liver Transplantation in Small Infants: Outcomes From a High‐Volume Center

**DOI:** 10.1111/petr.70456

**Published:** 2026-09-08

**Authors:** Helena Linge, Eva‐Doreen Pfister, Vanessa Muth, Sahar Arbabzadah, Jan Forner, Lisa Felgendreff, Nicolas Richter, Markus Quante, Moritz Schmelzle, Ulrich Baumann, Philipp Felgendreff, Cornelius J. Van Beekum

**Affiliations:** ^1^ Department of General, Visceral and Transplant Surgery Hannover Medical School Hannover Germany; ^2^ Clinic for Pediatric Kidney, Liver, and Metabolic Diseases and Neuropediatrics Hannover Medical School Hannover Germany

**Keywords:** graft survival, infant, liver transplantation, living donors, pediatrics

## Abstract

**Background:**

Graft selection in small infants is challenging due to size mismatch and the associated risk of vascular or biliary complications.

**Methods:**

In this retrospective single‐center study, 57 pediatric liver transplants using left lateral segment grafts performed between 2019 and 2024 were analyzed and compared by recipient weight (< 10 kg vs. ≥ 10 kg).

**Results:**

Overall graft survival was 88% in the < 10 kg group and 96% in the ≥ 10 kg group. Patient survival at 1, 2, and 4 years was similar between groups (*p* = 0.500). The graft‐to‐recipient weight ratio was significantly higher in smaller recipients (4.64% vs. 2.37%, *p* < 0.05), and hepatic artery thrombosis occurred only in this group (9%). Biliary complications were comparable between cohorts (27% vs. 30%). Acute cellular rejection (57% vs. 12%, *p* < 0.001) and 90‐day readmissions (57% vs. 27%, *p* = 0.022) were significantly more common in children weighing ≥ 10 kg.

**Conclusion:**

In this selected cohort, left lateral segment transplantation was associated with excellent outcomes across weight groups. Acceptable results in small infants were achieved despite higher GRWR in a specialized setting, supporting LLS grafts as an important option to improve access to timely pediatric transplantation.

## Graphical abstract

Left lateral segment liver transplantation achieved excellent graft and patient survival even in infants under 10 kg. Despite higher graft to recipient weight ratios and increased vascular complexity, outcomes remained comparable to larger children when transplantation was performed in a specialized high‐volume center.

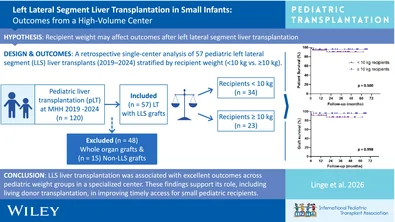

**Abbreviations**
ABOi: ABO‐incompatible
ARDS: acute respiratory distress syndrome
BA: biliary atresia
BMI: body mass index
CD: Clavien‐Dindo
CIT: cold ischemia time
CT: computer tomography
DDLT: deceased donor liver transplantation
GRWR: graft‐to‐recipient weight ratio
HAT: hepatic artery thrombosis
INR: international normalized ratio
IQR: interquartile range
LDLT: living donor liver transplantation
LLS: left lateral segment
MRI: magnetic resonance imaging
PELD: pediatric end‐stage liver disease
PLT: pediatric liver transplantation
PTCD: percutaneous transhepatic cholangiography drainage
PVT: portal vein thrombosis
RBC: red blood cell
rt‐PA: recombinant tissue plasminogen activator
SD: standard deviation
UNOS: United Network for Organ Sharing
WIT: warm ischemia time

## 1 Introduction

Pediatric liver transplantation (PLT) is a life‐saving treatment for children with end‐stage liver disease but remains particularly challenging in infants weighing less than 10 kg. In neonates and very small children, the availability of size‐appropriate whole‐organ grafts is limited due to anatomical constraints and overall donor shortage. The use of partial liver grafts has made transplantation possible in this vulnerable group [1].

Left lateral segment (LLS) grafts, comprising segments II and III, are the most used graft type in infants and can be procured from both deceased (DDLT) and living donors (LDLT) [2]. Their use has allowed centers to overcome the limitations posed by graft size mismatch and organ availability. In pediatric LDLT, a suitable living donor provides a portion of their liver for transplantation to the child. Both LDLT and DDLT involve different but equally complex steps in donor assessment, surgical planning, and technique.

Matching donor grafts to pediatric recipients, especially when using split or living donor grafts, depends strongly on the surgical team's experience and institutional practice. Pediatric liver transplantation is a highly specialized procedure performed at few centers, but with increasing experience, survival rates continue to improve, making it a highly effective treatment for end‐stage liver disease [3].

While various factors such as the underlying diagnosis, the patient's condition at the time of transplantation, graft type, donor status, and ischemia time influence outcomes, there is a lack of consensus‐based data on donor‐recipient matching, surgical technique, and postoperative management in small infants. Although some studies have addressed kidney injury [4], long‐term outcomes [5, 6], split liver techniques [1, 7], and intraoperative lactate clearance as a predictor of outcome [8], detailed data on infants under 10 kg are sparse. ELTR data confirm that long‐term graft survival in pediatric liver transplantation has steadily improved, with particularly favorable outcomes in children under 12 years [9]. However, biliary leaks and strictures remain common postoperative complications, with incidences reported as high as 45% in some cohorts [10, 11]. Reported rates of hepatic artery thrombosis range from 2% to 4%, and portal vein thrombosis from 4% to 16% [10, 12, 13]. Despite growing interest in graft‐to‐recipient size mismatch, clear threshold values in pediatric liver transplantation, especially among recipients weighing less than 10 kg, are still lacking.

This single‐center, retrospective study aims to evaluate postoperative complications, graft outcomes, and patient survival in pediatric recipients weighing less than 10 kg compared with those ≥ 10 kg undergoing liver transplantation with a left lateral segment graft. By analyzing weight‐based differences, we aim to contribute additional evidence to support risk assessment and graft selection in small pediatric recipients.

## 2 Patients and Methods

### 2.1 Study Design

Pediatric liver transplants performed at Hannover Medical School between 2019 and 2024 were reviewed. A total of 120 liver transplants in patients under 18 years of age were identified. The following cases were excluded from analysis: recipients of monosegmental grafts (*n* = 1), deceased donor grafts with size reduction procedures (e.g., right posterior sectionectomy; *n* = 5), extended right lobe grafts (*n* = 2), whole liver grafts (*n* = 48), re‐transplantations (*n* = 6), and combined liver‐kidney transplants (*n* = 1). This resulted in a final study cohort of 57 patients who received either postmortem or living donor left lateral segment (segments II/III) grafts. Outcomes assessed included graft and patient survival, postoperative complications, graft function, and hospital re‐admissions. Patients were divided into two cohorts based on recipient body weight (< 10 kg vs. ≥ 10 kg).

Data were obtained from electronic medical records and included demographic information, surgical and anesthetic details, hospital stay, and clinical outcomes. Surgical complications were classified according to the Clavien‐Dindo system. Vascular and biliary complications were assessed separately, with biliary complications categorized as bile leaks, anastomotic strictures, or non‐anastomotic strictures.

Short‐ and long‐term outcomes were compared between recipients < 10 kg and ≥ 10 kg, using standardized follow‐up intervals (3 months and 1 year).

### 2.2 Statistical Analysis

Demographic data, surgical details, postoperative outcomes, and complications were analyzed according to the respective variables. Categorical variables were presented as counts (*n*) and percentages (%) and analyzed using the Chi‐square test. Continuous variables were reported as medians with interquartile ranges (IQR) if not normally distributed and analyzed using the Mann–Whitney *U* test. Normally distributed variables were presented as means with standard deviations (SD) and analyzed using the independent *t*‐test (with Levene's test for variance equality). A *p*‐value of < 0.05 was considered statistically significant. Kaplan–Meier analysis was performed to compare graft and patient survival. No censoring was applied due to complete follow‐up data.

All statistical analyses were conducted using IBM SPSS Statistics (version 22.0, IBM Corporation, Armonk, NY, USA) and GraphPad Prism (GraphPad Software, San Diego, CA, USA). A *p*‐value < 0.05 was considered statistically significant.

### 2.3 Ethics Statement

Informed consent for anonymous use of clinical data for research purposes was obtained from all parents or caregivers. All patient data were anonymized prior to analysis. According to our institutional ethics committee guidelines, approval was given (11651_BO_K_2024).

### 2.4 Standard Operative Technique for Left Lateral Segment Liver Transplantation in Pediatric Recipients

All pediatric liver transplants were performed at Hannover Medical School using a standardized cava‐preserving technique by the same surgical team, with perioperative care provided by experienced pediatric intensivists and gastroenterologists. After induction of general anesthesia, a combined upper midline and transverse laparotomy was used. Adhesiolysis was limited, and the hilum was dissected to prepare the portal vein, hepatic artery, and bile duct or Kasai loop. Hepatectomy was completed with preservation of the inferior vena cava, and the left lateral segment graft (II/III) was implanted. Venous outflow was reconstructed via end‐to‐side anastomosis, and portal vein reconstruction was performed end‐to‐end, using interposition grafts or renal vein ligation when required. Arterial anastomosis was completed end‐to‐end, with branch‐patch or aortic interposition grafts used selectively. Vascular and biliary anastomoses were performed with absorbable monofilament sutures. In unstable patients, temporary abdominal closure with planned second‐look surgery was used, and biliary drainage was achieved by Roux‐en‐Y hepaticojejunostomy. Primary abdominal closure was performed when feasible; otherwise, temporary closure with mesh or staged fascial repair was applied.

### 2.5 Perioperative Anticoagulation Protocol

Perioperative anticoagulation was administered to all recipients. Intraoperative unfractionated heparin was given at reperfusion, followed by a continuous heparin infusion of 200 IU/kg/day initiated postoperatively, starting at approximately 100 IU/kg/day and titrated based on coagulation parameters. Heparin was only administered when PTT was below 45 s and INR below 2.5, and was continued for 1 week post‐transplant. Antithrombin III levels were maintained above 60% for the first three postoperative days. Aspirin was added at 3 mg/kg/day (maximum 75 mg) once platelet counts exceeded 50 000/μl and continued for a minimum of 3 months post‐transplant. Clopidogrel and Iloprost were reserved for special indications only. Doppler ultrasound monitoring of hepatic artery and portal vein flow was performed daily in all patients. No differences in anticoagulation management were applied between ABO‐incompatible and ABO‐compatible recipients. This protocol was applied to both weight groups.

## 3 Results

### 3.1 Recipients

The significant differences between the two weight groups (< 10 kg vs. ≥ 10 kg) presented in Table 1 were primarily related to recipient characteristics and pre‐transplant conditions. Recipients in the < 10 kg group were significantly younger and smaller in terms of age, body weight, and height. Sex distribution also differed between groups. Additionally, significant differences were observed in donor‐recipient sex matching and the frequency of previous Kasai procedures, both being more common in the < 10 kg group. Regarding recipient diagnoses, biliary atresia was more prevalent in smaller children, while other cholestatic diseases were more frequent in the ≥ 10 kg group. Furthermore, the PELD score was significantly higher in the < 10 kg group, reflecting greater disease severity at the time of transplantation.

**TABLE 1:** Recipient demographic data.

Recipient demographics	Total	Recipients < 10 kg	Recipients ≥ 10 kg	*p*
Recipients, *n* (%)	57 (100)	34 (60)	23 (40)	
Sex, *n* (%)				
Male	33 (58)	15 (44)	18 (78)	0.014
Female	24 (42)	19 (56)	5 (22)	
Age at transplant (months), median (IQR)	14 (17)	7.5 (3)	34 (40)	< 0.001
Body weight (kg), median (IQR)	8 (6)	7 (2)	15 (4)	< 0.001
Height (m), median (IQR)	0.7 (0.2)	0.7 (0.1)	1 (0.2)	< 0.001
Sex donor match, *n* (%)				
Female recipient/male donor	17 (30)	12 (35)	5 (22)	0.038
Female recipient/female donor	7 (12)	7 (21)	0 (0)	
Male recipient/male donor	20 (35)	10 (29)	10 (44)	
Male recipient.female donor	13 (23)	5 (15)	8 (35)	
Pre‐transplantation conditions, *n* (%)				
Portal vein thrombosis	5 (9)	2 (6)	3 (13)	0.348
Congenital heart disease	8 (14)	4 (12)	4 (17)	0.549
Hepatopulmonary syndrome	2 (4)	2 (6)	0 (0)	0.236
Kasai procedure	26 (46)	22 (65)	4 (17)	< 0.001
Pre transplantation dialysis	2 (4)	1 (3)	1 (4)	0.777
Recipient diagnosis, *n* (%)				
Biliary Atresia (BA)	26 (46)	24 (71)	2 (9)	< 0.001
Other cholestatic diseases	9 (16)	1 (3)	8 (35)	< 0.001
Metabolic disorders	7 (12)	4 (12)	3 (13)	1.000
Acute liver failure	5 (9)	1 (3)	4 (17)	0.067
Liver tumors	5 (9)	1 (3)	4 (17)	0.067
Other indications	5 (9)	3 (9)	2 (9)	1.000
Pre‐transplantation blood results				
PELD score, median (IQR)	28 (11)	30 (0)	28 (12)	0.005
Serum sodium (mmol/L), mean (SD)	131 (32)	128 (37)	139 (5)	0.372
INR, mean (SD)	1.6 (0.8)	1.6 (0.7)	1.69 (0.9)	0.510
Total bilirubin (μmol/L), mean (SD)	263 (200)	316 (206)	143 (127)	0.036
Creatinine (μmol/L), mean (SD)	25 (36)	19 (7)	43 (71)	0.090
Platelet count (G/L), mean (SD)	156 (126)	148 (133)	127 (104)	0.423
Serum albumin (g/L), mean (SD)	32 (9)	31 (9)	34 (6)	0.493

*Note:* Categorical data are shown as *n* (%) and tested with Chi‐square. Non‐normal continuous data are shown as median (IQR) and tested with Mann–Whitney *U*. Normal data are shown as mean (SD) and tested with independent *t*‐test.

Abbreviations: IQR, interquartile range; SD, standard deviation.

### 3.2 Donor and Graft Characteristics

Donor characteristics, including sex distribution, age, and BMI, were comparable across weight groups, with living donors being generally older and having a slightly higher BMI than deceased donors. ABO‐incompatible transplantations were more frequently performed in recipients under 10 kg, particularly using living donors. No significant association between donor‐recipient sex matching and survival was observed in our cohort. GRWR was significantly higher in recipients under 10 kg, reflecting smaller body weight despite lighter grafts, while absolute graft weight was greater in the ≥ 10 kg group (Table 2).

**TABLE 2:** Donor characteristics.

Donor characteristics	Total	Recipients < 10 kg	Recipients ≥ 10 kg	*p*
Sex, *n* (%)				
Male	37 (65)	22 (65)	15 (65)	0.968
Female	20 (35)	12 (35)	8 (35)	
Age (years), median (IQR)	29 (12)	30 (11)	29 (14)	0.287
BMI kg/m^2^, median (IQR)	24 (5)	24 (6)	24 (4)	0.130
Donor type, *n* (%)				
LDLT	23 (40)	21 (62)	2 (9)	< 0.001
DDLT	34 (60)	13 (38)	21 (91)	
AB0‐incompatible, *n* (%)	8 (14)	7 (21)	1 (4)	0.083
Graft characteristics				
Weight (g), median (IQR)	306 (93)	289 (69)	345 (83)	0.007
GRWR (%), median (IQR)	3.39 (3)	4.64 (2)	2.37 (1)	< 0.001

*Note:* Categorical data are shown as *n* (%) and tested with Chi‐square. Non‐normal continuous data are shown as median (IQR) and tested with Mann–Whitney *U*. Normal data are shown as mean (SD) and tested with independent *t*‐test.

Abbreviations: DDLT, deceased donor liver transplantation; GRWR, graft‐to‐recipient weight ratio; LDLT, living donor liver transplantation.

### 3.3 Surgical and Technical Details

There were no significant differences between the weight groups in operative time, warm ischemia time, intraoperative blood loss, or transfusion requirements as outlined in Table 3. However, cold ischemia time was significantly longer in recipients ≥ 10 kg. Prior abdominal surgery did not significantly impact operative duration.

**TABLE 3:** Intraoperative parameters, including surgical and technical details.

Intraoperative parameters	Total	Recipients < 10 kg	Recipients ≥ 10 kg	*p*
Surgical details				
Operative time (min), median (IQR)	255 (83)	265 (78.3)	242 (78)	0.313
Cold ischemia time (min), mean (SD)	495 (194)	423 (182)	601 (161)	0.002
Warm ischemia time (min), mean (SD)	52 (12)	52 (14)	52 (10)	0.484
Intraoperative blood loss (mL), mean (SD)	590 (435)	594 (471)	583 (387)	0.957
Red blood cell transfusion (units), median (IQR)	2 (1)	2 (0.75)	2 (1)	0.671
Technical details				
Number of bile duct anastomosis, *n* (%)				0.366
1	49 (86)	27 (79)	22 (96)	
2	6 (11)	5 (15)	1 (4)	
3	1 (2)	1 (3)	0 (0)	
unknown	1 (2)	1 (3)	0 (0)	
Hepaticojejunostomy, *n* (%)	57 (100)	34 (100)	23 (100)	^a^
Use of interposition grafts, *n* (%)	16 (28)	10 (29)	1 (4)	0.037
Portal flow modulation, *n* (%)	2 (4)	2 (6)	0 (0)	0.236

*Note:* Categorical data are shown as *n* (%) and tested with Chi‐square. Non‐normal continuous data are shown as median (IQR) and tested with Mann–Whitney *U*. Normal data are shown as mean (SD) and tested with independent *t*‐test.

^a^ Indicates no significance was calculated, due to only one variable available.

Hepaticojejunostomy was the standard biliary reconstruction technique. Portal vein interposition grafts were used more frequently in the < 10 kg group (10 cases) compared to the ≥ 10 kg group (1 case), although overall utilization remained low. Portal flow modulation, such as ligation of a splenorenal shunt or the left renal vein, was applied exclusively in two < 10 kg recipients to increase portal inflow in the context of higher GRWR.

### 3.4 Early Postoperative Complications (< 90 Days)

Postoperative recovery, assessed by median hospital stay, did not differ significantly between groups (52 days [SD 23] vs. 52 days [SD 42]; *p* = 0.598, Levene's test + independent *t*‐test). However, readmissions within 90 days were significantly more frequent among larger recipients (9 [27%] vs. 13 [57%]; *p* = 0.022, Chi‐square test). Postoperative dialysis was required in 3 patients (9%) in the < 10 kg group, compared to 1 patient (4%) in the ≥ 10 kg group. Although slightly more frequent in smaller recipients, the difference was not statistically significant and overall dialysis requirement remained low in both cohorts.

Complications were classified using the Clavien‐Dindo system. Both weight groups experienced a range of minor to moderate complications, most frequently Grade II and IIIA. Severe complications (Grades IIIB, IV, and V) were rare in both groups, and there were no significant differences in the overall severity or frequency of complications during the first 3 months after transplantation.

### 3.5 Postoperative Complications at 1 Year

Late complications, assessed at one‐year post‐transplantation, were infrequent in both groups. The distribution of Clavien‐Dindo showed no significant differences, and severe complications (Grade IVA or higher) were rare (Table 4).

**TABLE 4:** Clavien‐Dindo classification grade < 90 days and at 1 year follow‐up after transplantation.

Clavien‐Dindo‐classification	Total	Recipients < 10 kg	Recipients ≥ 10 kg	*p*
Complications < 90 days after transplant, *n* (%)				0.727
Grade II	10 (18)	7 (21)	3 (13)	
Grade IIIA	16 (28)	9 (27)	7 (30)	
Grade IIIB	15 (26)	8 (24)	7 (30)	
Grade IVA	5 (9)	3 (9)	2 (9)	
Grade IVB	2 (4)	2 (6)	0 (0)	
Complications at 1 year post transplant, *n* (%)				0.072
Grade II	7 (12)	7 (21)	0 (0)	
Grade IIIA	7 (12)	4 (12)	3 (13)	
Grade IIIB	4 (7)	1 (3)	3 (13)	
Grade IVA	1 (2)	1 (3)	0 (0)	
Grade IVB	1 (2)	1 (3)	0 (0)	

*Note:* Categorical data are shown as *n* (%) and tested with Chi‐square.

### 3.6 Vascular Complications

Hepatic artery thrombosis (HAT) occurred exclusively in the < 10 kg group, affecting 3 patients (9%). Two of these patients also developed portal vein thrombosis (PVT), resulting in graft loss and requiring re‐transplantation; one patient died following complications. The third patient was successfully treated with systemic thrombolysis using rt‐PA. No cases of HAT were observed in the ≥ 10 kg group.

PVT was similarly more frequent in the < 10 kg group, with five cases (15%) including the two re‐transplantation cases mentioned above, compared to just one case (4%) in the ≥ 10 kg group. Among the remaining three patients in the < 10 kg group, two were treated with anticoagulation and radiological recanalization, and one underwent surgical embolectomy. The single case in the ≥ 10 kg group was managed with anticoagulation and radiological intervention.

### 3.7 Biliary Complications

Biliary complications were classified into bile leaks, anastomotic strictures, and non‐anastomotic strictures. The overall incidence was comparable between the two weight groups. In the < 10 kg cohort, nine patients (27%) developed biliary complications, with anastomotic strictures being the most common (*n* = 6). Two patients experienced bile leaks, and one patient developed a non‐anastomotic stricture. In the ≥ 10 kg group, biliary complications occurred in seven patients (30%), including four bile leaks and three anastomotic strictures. Management strategies comprised observation, radiologic drainage, and surgical revision. Radiologic drainage was performed in five patients (three in the < 10 kg group and two in the ≥ 10 kg group), while surgical revision was required in nine cases (4 < 10 kg, 5 ≥ 10 kg). One patient in the < 10 kg group was managed conservatively without intervention.

When primary interventions failed, secondary interventions were required in eight cases. Repeat radiologic drainage was performed in three patients (two in the < 10 kg, 1 in the ≥ 10 kg group), one patient in the ≥ 10 kg group underwent PCTD, and four patients (two in each group) required reoperation. There was no statistically significant difference between the groups in the need for either primary or secondary intervention. One patient in the ≥ 10 kg group experienced treatment failure following secondary interventions due to persistent anastomotic biliary strictures, severe intrahepatic cholestasis, multiple PTCD placements, and the need for additional drainage after recurrent leakage at the biliary anastomosis.

### 3.8 Other Surgical Complications

Bowel perforation occurred in two patients in the < 10 kg group, one with a prior Kasai operation who also developed intra‐abdominal bleeding, and one without previous surgery, as well as in one patient in the ≥ 10 kg group, who also experienced intra‐abdominal bleeding without prior abdominal surgery. Intra‐abdominal bleeding occurred in four patients in each group (including those with perforation), with no significant difference in frequency between groups.

### 3.9 Acute Cellular Rejection

There was a markedly higher incidence of biopsy‐proven acute cellular rejection in the ≥ 10 kg group, affecting 13 patients (57%) compared to only 4 patients (12%) in the < 10 kg group (*p* < 0.001).

### 3.10 Graft Survival

**FIGURE 1:**
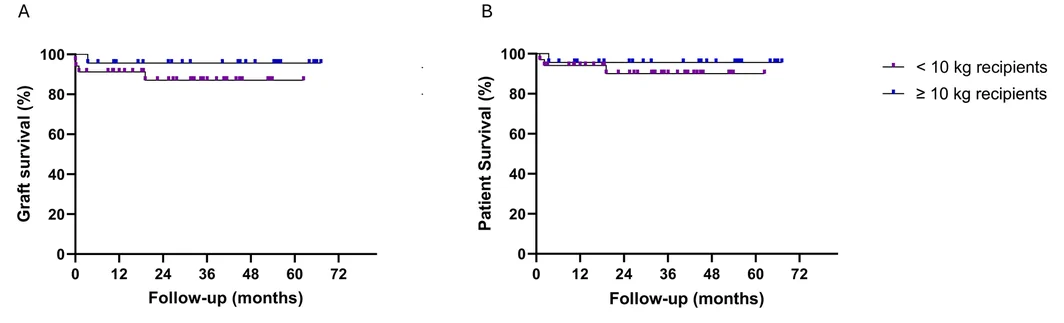
Kaplan–Meier curves for (A) graft survival (*p* = 0.998) and (B) patient survival (*p* = 0.500) by weight group in pediatric recipients (log‐rank test).

Graft survival was excellent in both groups, with a slightly higher rate in children ≥ 10 kg. In the < 10 kg group, 4 out of 34 patients experienced graft loss (observed graft survival: 88%), whereas only one graft loss occurred in the ≥ 10 kg group (96%). Estimated graft survival at 1, 2, and 4 years post‐transplant was 91%, 87%, and 87% respectively in the < 10 kg group, compared to 100% at 1 year and 96% at 2 and 4 years in the ≥ 10 kg group (Figure 1A). The difference was not statistically significant (*p* = 0.998). Of the four patients with graft loss, two underwent re‐transplantation, one of whom subsequently died despite the second transplant. No re‐transplantations were performed in recipients ≥ 10 kg. The single graft loss in the ≥ 10 kg group was due to multiorgan failure caused by the underlying disease, as discussed below. Although limited by sample size, these findings suggest a higher likelihood of technical complications in smaller recipients, particularly vascular thrombosis. Still, long‐term graft function remained favorable in both cohorts.

### 3.11 Patient Survival

Patient survival had a similar pattern. Three patients died in the < 10 kg group during the follow‐up period due to severe post‐transplant complications. One patient died 1 month after transplantation following hemorrhagic shock, multiple reoperations, multiorgan failure, status epilepticus with cardiac arrest, and terminal kidney failure. Another developed hepatic artery thrombosis requiring revision surgery, followed by portal vein thrombosis, and underwent high‐urgency re‐transplantation; this patient later died due to sepsis and secondary biliary cirrhosis. The third patient died 19 months post‐transplant from sepsis with multiorgan failure, despite antibiotic therapy. In the ≥ 10 kg group, one patient died 3.5 months post‐transplant from therapy‐refractory sepsis, ARDS, and chemotherapy‐related complications. The underlying diagnosis was Stage IV hepatoblastoma with portal vein thrombosis, lung metastases, and a complex pre‐transplant course including severe tumor bleeding, multiple abdominal surgeries with open abdomen, tracheostomy, and ongoing chemotherapy. At 1‐, 2‐, and 4‐year post‐transplant, patient survival in the < 10 kg group was 94%, 90% and 90% respectively, compared to 96% at 1‐, 2‐, and 4‐year time points in the ≥ 10 kg group (Figure 1B). No statistically significant difference was observed between groups (*p* = 0.500).

### 3.12 Donor Type Subgroup Analysis

A subgroup analysis comparing outcomes by donor type was performed across the full cohort (Table 5). LDLT recipients showed significantly higher rates of portal vein thrombosis (22% vs. 3%, *p* = 0.034). LDLT recipients had significantly shorter cold ischemia time (283 vs. 630 min, *p* < 0.001) and higher GRWR (4.29% vs. 2.85%, *p* = 0.005), as expected for living donor grafts. Acute cellular rejection was lower in the LDLT group (17% vs. 41%, *p* = 0.083). Patient survival, graft loss, hepatic artery thrombosis, biliary complications, and 90‐day readmission rates were comparable between donor types.

**TABLE 5:** Donor type subgroup analysis: Outcomes in LDLT versus DDLT recipients.

Outcomes	Total (*n* = 57)	LDLT (*n* = 23)	DDLT (*n* = 34)	*p*
Graft characteristics				
Cold ischemia time, min, median (IQR)	528 (341)	283 (98)	630 (121)	< 0.001
GRWR, %, median (IQR)	3.39 (2.68)	4.29 (1.95)	2.85 (1.74)	0.005
Major complications				
Hepatic artery thrombosis, *n* (%)	3 (5)	3 (13)	0	0.061
Portal vein thrombosis, *n* (%)	6 (11)	5 (22)	1 (3)	0.034
Biliary complications, *n* (%)	17 (30)	6 (26)	11 (32)	0.770
Acute cellular rejection, *n* (%)	18 (32)	4 (17)	14 (41)	0.083
90‐day readmission, *n* (%)	22 (39)	7 (30)	15 (44)	0.407
Survival				
Patient survival, *n* (%)	52 (91)	22 (96)	31 (91)	0.641
Graft loss, *n* (%)	4 (7)	2 (9)	2 (6)	1.000

*Note:* Categorical data are shown as *n* (%) and tested with Fisher's exact test. Non‐normal continuous data are shown as median (IQR) and tested with Mann–Whitney *U*. Patient survival reported as overall survival.

Abbreviations: CIT, cold ischemia time; DDLT, deceased donor liver transplantation; GRWR, graft‐to‐recipient weight ratio; LDLT, living donor liver transplantation.

## 4 Discussion

This five‐year single‐center analysis confirms that pediatric liver transplantation using left lateral segment grafts is both safe and effective in our center, even in infants weighing less than 10 kg. Although this group faces technical and physiological challenges, such as small vessel size and high GRWR, outcomes were excellent and comparable to larger recipients. These findings reflect results achieved in a highly specialized, high‐volume center and should be interpreted in the context of center‐specific expertise and perioperative strategies. These results support the feasibility of liver transplantation in the smallest patients when performed at experienced, high‐volume centers.

Beyond graft size alone, other donor and graft‐related factors such as cold ischemia time, graft steatosis, and center experience can also influence transplant outcomes. Several studies have shown that graft size should not be the only factor when selecting donors or estimating risk [14, 15]. A pilot project from Germany reported favorable outcomes with reduced‐size grafts in pediatric recipients, including infants, although no specific GRWR thresholds were defined for children under 10 kg [16]. While a GRWR below 0.6% has been associated with increased risk of early graft loss in adult recipients [17] these thresholds cannot be directly applied to the pediatric setting, where smaller body size and higher metabolic demands necessitate proportionally larger grafts to ensure adequate function [18, 19]. In pediatric cases, a GRWR above 2.5% may lead to delayed abdominal closure but does not appear to affect long‐term outcomes [20, 21].

In our cohort, one of the main findings in the < 10 kg group was a clearly higher GRWR, often over 4%. Although high GRWR and graft‐recipient mismatch are considered risk factors for vascular or biliary complications, we did not observe a statistically significant association in this selected cohort. This does not mean that high GRWR is without risk; rather, careful graft selection, surgical technique, and perioperative management may reduce its effects. At our center, the selection of the donation after brain death graft is based on a combination of the estimated graft weight relative to the recipient's body weight, an assessment of parenchymal quality, anatomical variations, and the anticipated position of the graft relative to the available intra‐abdominal space in the recipient. For living donor grafts, preoperative volumetric CT scan combined with an MRI is routinely performed to estimate graft weight and ensure an adequate remnant liver volume for the donor. In cases where a high GRWR is anticipated, temporary abdominal closure is planned proactively, and portal flow modulation is considered intraoperatively. These findings are in line with previous reports and suggest that acceptable outcomes can be achieved with large‐for‐size grafts in infants under specific conditions. Therefore, our data did not allow for the identification of a definitive GRWR cut‐off. The need for postoperative dialysis was higher in the < 10 kg group, reflecting a higher preoperative morbidity in this cohort. The proportion of living donor liver transplantation was significantly higher in this group, mainly due to urgent clinical indications and the limited availability of size‐matched deceased donor grafts. While DDLT is generally preferred, LDLT remains essential in smaller children, where timely access to suitable organs is often not possible.

LDLT is essential for timely access in small, high‐risk recipients, especially given the rarity of size matched deceased donor organs, reflecting its growing importance in the German transplant system. The higher use of LDLT in smaller recipients introduces an inherent selection bias, as it allows for preoperative planning of graft size, assessment of abdominal domain, and optimization of venous outflow. As expected, DDLT was associated with longer CIT and, interestingly, higher rejection rates. However, neither affected graft or patient survival compared to the LDLT cohort. In the LDLT cohort, the higher rate of PVT did not impact graft or patient survival and likely reflects the technical complexity of smaller vessels rather than a donor‐type–related effect. This is in line with findings from the prospective ChilSFree study, which identified LDLT as an independent predictor of rejection‐free survival during the first year, even after adjusting for confounders [22]. LDLT offers advantages such as reduced ischemic injury and improved early graft outcomes, with survival rates comparable to split deceased donor grafts, though it may carry a higher risk of vascular complications [23].

Most cases of graft loss occurred in the < 10 kg group; however, the overall number was low and primarily related to early vascular complications, particularly arterial issues, which were more likely due to the technical complexity of anastomosing very small hepatic arteries rather than graft size or donor type alone. The only graft loss in the ≥ 10 kg group resulted from treatment‐related complications of hepatoblastoma and subsequent multiorgan failure due to sepsis. As expected, vascular complications such as hepatic artery and portal vein thrombosis were more common in smaller recipients. However, most cases were successfully treated with radiologic or surgical intervention and did not affect overall survival. Reasons for vascular complications might be portal hypoperfusion and arterial micro anastomosis or sequential thrombosis of both vessels. Techniques such as portal flow modulation and interposition grafts were used when needed, based on the surgeon's judgment, to reduce the risk of portal vein thrombosis.

Acute cellular rejection was significantly more frequent in the ≥ 10 kg group, possibly reflecting age‐related differences in immune response, metabolism, and donor matching, as well as the lower use of LDLT in this group. Living donor grafts are associated with shorter cold ischemia times and better early graft function, which may reduce immunogenicity [22, 24, 25]. These findings suggest a lower risk of early rejection with LDLT. Additionally, variations in immunosuppressive protocols across centers may influence rejection rates. At our center, we routinely use a steroid‐free protocol (intraoperative steroid bolus only, followed by tacrolimus monotherapy), which differs from many pediatric centers and may contribute to the higher observed rates of acute cellular rejection. In cases of ABO‐incompatible transplantation, however, we do include primary steroid therapy. The use of both ABO‐compatible and ABO‐incompatible grafts, together with a balanced application of living and deceased donors, reflects a flexible and practical transplant strategy. The low rate of re‐transplantation and manageable complication rate support the value of standardized surgical techniques and consistent perioperative care, even in high‐risk patients.

Several limitations should be acknowledged. This was a retrospective, single‐center study with a relatively small sample size, particularly in the ≥ 10 kg group. Cohort selection, including the exclusion of monosegment grafts and reduced grafts, may have introduced additional bias. Donor type distribution was highly imbalanced between weight groups and represents a significant confounder limiting the interpretation of between‐group comparisons. A fully stratified subgroup analysis by both weight and donor type was not feasible given the available sample size. Despite these limitations, this study provides a comprehensive overview of LLS transplantation in a highly center‐specific setting. To our knowledge, it is among the first to demonstrate comparable outcomes in children weighing less than 10 kg compared to larger recipients. Future prospective multicenter studies are needed to validate these findings, define safe GRWR ranges, and evaluate the impact of immunosuppressive protocols in infants.

## 5 Conclusion

Liver transplantation using left lateral segment grafts is safe and effective even in infants under 10 kg when performed in experienced centers. In this selected cohort, acceptable outcomes were observed despite higher GRWR, without a demonstrable increase in vascular or biliary complications. These findings should not be interpreted as evidence that high GRWR is universally well tolerated, but rather that its impact may be modulated by graft selection, surgical expertise, and perioperative management. Although smaller recipients showed more vascular events, outcomes remained excellent with timely management. Limited access to size‐matched deceased organs underlines the importance of living donor transplantation, especially in high‐risk infants. Sustained success in this group requires interdisciplinary expertise, standardized protocols, and experienced and high‐volume centers. The results of this study may not be generalizable to centers with lower pediatric transplant volumes or limited access to LDLT. Expanding pediatric LDLT programs will be essential to meet future needs and improve care for this vulnerable population.

## Disclosure

M.S. reports consulting and/or research support from Merck Serono GmbH, Bayer AG, ERBE Elektromedizin GmbH, Amgen Inc., AstraZeneca, Takeda Pharmaceutical Limited, Bowa Medical, Corza Medical, Medtronic GmbH, Johnson & Johnson Medical GmbH, Intuitive Surgical Inc., Chiesi GmbH, OrganOx, and Baxter International Inc.

## Ethics Statement

Approval of the use of patient data in this study was obtained from the local ethics committee (11651_BO_K_2024).

## Conflicts of Interest

The authors declare no conflicts of interest.

## Data Availability

The data that support the findings of this study are available from the corresponding author upon reasonable request.
